# What is known about the effects of vitamin D in neuropsychiatric lupus?

**DOI:** 10.1186/s42358-023-00344-w

**Published:** 2024-01-02

**Authors:** Thaís Evelyn Karnopp, Vinicius da Silva Freitas, Andressa Leite Di Domenico, Gustavo Flores Chapacais, Natália Garcia dos Santos, Eduarda Correa Freitas, Andrese Aline Gasparin, Odirlei André Monticielo

**Affiliations:** 1 Laboratory of Autoimmune Diseases, Division of Rheumatology, Hospital de Clínicas de Porto Alegre, Centro de Pesquisas Experimentais, Universidade Federal Do Rio Grande Do Sul, Rua Ramiro Barcelos, 2350, Sala 12109, Porto Alegre, 90035‑003 Brazil; 2 Postgraduate Program in Medical Sciences, Universidade Federal do Rio Grande do Sul, Porto Alegre, RS Brazil; 3 Division of Rheumatology, Department of Internal Medicine, Hospital de Clínicas de Porto Alegre, Universidade Federal Do Rio Grande Do Sul, Porto Alegre, Brazil; 4 Postgraduate Program in Biological Sciences: Pharmacology and Therapeutics, Porto Alegre, RS Brazil

**Keywords:** Systemic lupus erythematosus, Neuropsychiatric lupus, Vitamin D, Hypovitaminosis D, Vitamin D supplementation

## Abstract

Systemic lupus erythematosus (SLE) is an autoimmune disease that can affect several organs and systems. The central and/or peripheral nervous system can suffer from complications known as neuropsychiatric lupus (NPSLE). Studies have associated the manifestations of SLE or NPSLE with vitamin D deficiency. It has been shown that hypovitaminosis D can lead to cognition deficits and cerebral hypoperfusion in patients with NPSLE. In this review article, we will address the main features related to vitamin D supplementation or serum vitamin D levels with neuropsychiatric manifestations, either in patients or in animal models of NPSLE.

**Graphical abstract:**

**Figure:**
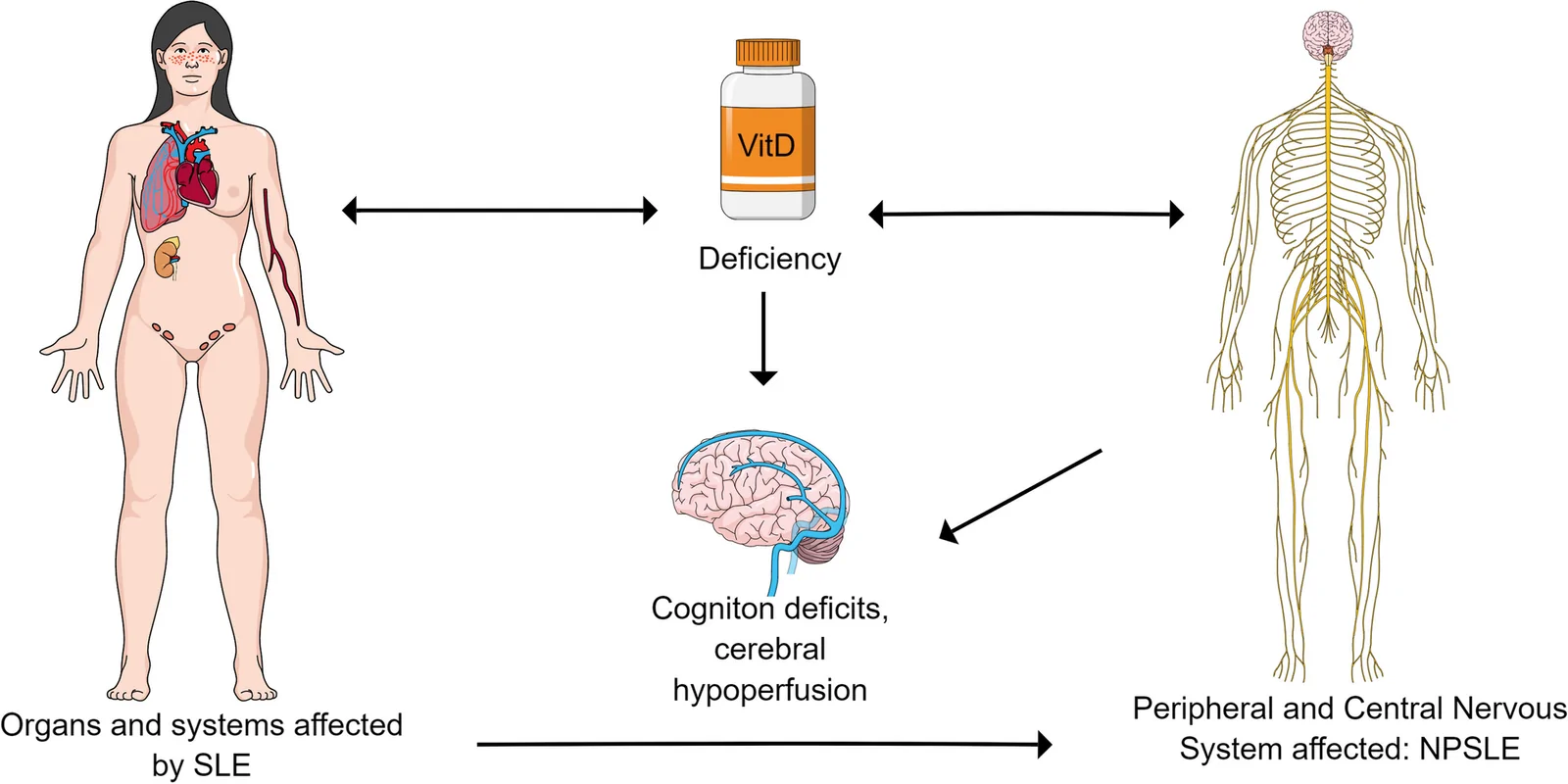

## Background

Systemic lupus erythematosus (SLE) is an autoimmune disease characterized by the production of autoantibodies and chronic inflammation. It can affect various organs and systems, such as the central nervous system (CNS) and/or the peripheral nervous system (PNS). When these are compromised, the consequent syndrome is known as neuropsychiatric lupus (NPSLE) [1, 2]. NPSLE is the second leading cause of morbidity and mortality in SLE patients [7]. Some of the observed clinical features are headache, mood disorder, seizures and psychosis [3].

Vitamin D (vit-D) low levels are frequently identified in autoimmune diseases, such as rheumatoid arthritis, type 1 diabetes mellitus and SLE [4–6]. In animal models of SLE, some studies have demonstrated that vit-D supplementation improves important outcomes like proteinuria, arthritis, skin lesions and survival [7, 8].

Vit-D, which acts in the immunomodulation of the innate and adaptive immune response, has also been investigated as an alternative therapy to control or minimize the clinical manifestations of SLE. Studies published in recent years demonstrated the importance of vit-D, not only in bone and calcium metabolism, but also in the regulation of the immune system, and in other tissues, such as the brain [9–11]. Furthermore, the deficiency of vit-D has been associated with the severity of clinical manifestations in patients with SLE [10, 11].

In this review, we examined studies related to hypovitaminosis D in patients with SLE and neuropsychiatric manifestations, as well as data regarding to vitamin D supplementation in animal models of NPSLE.

## Materials and methods

**Fig. 1:**
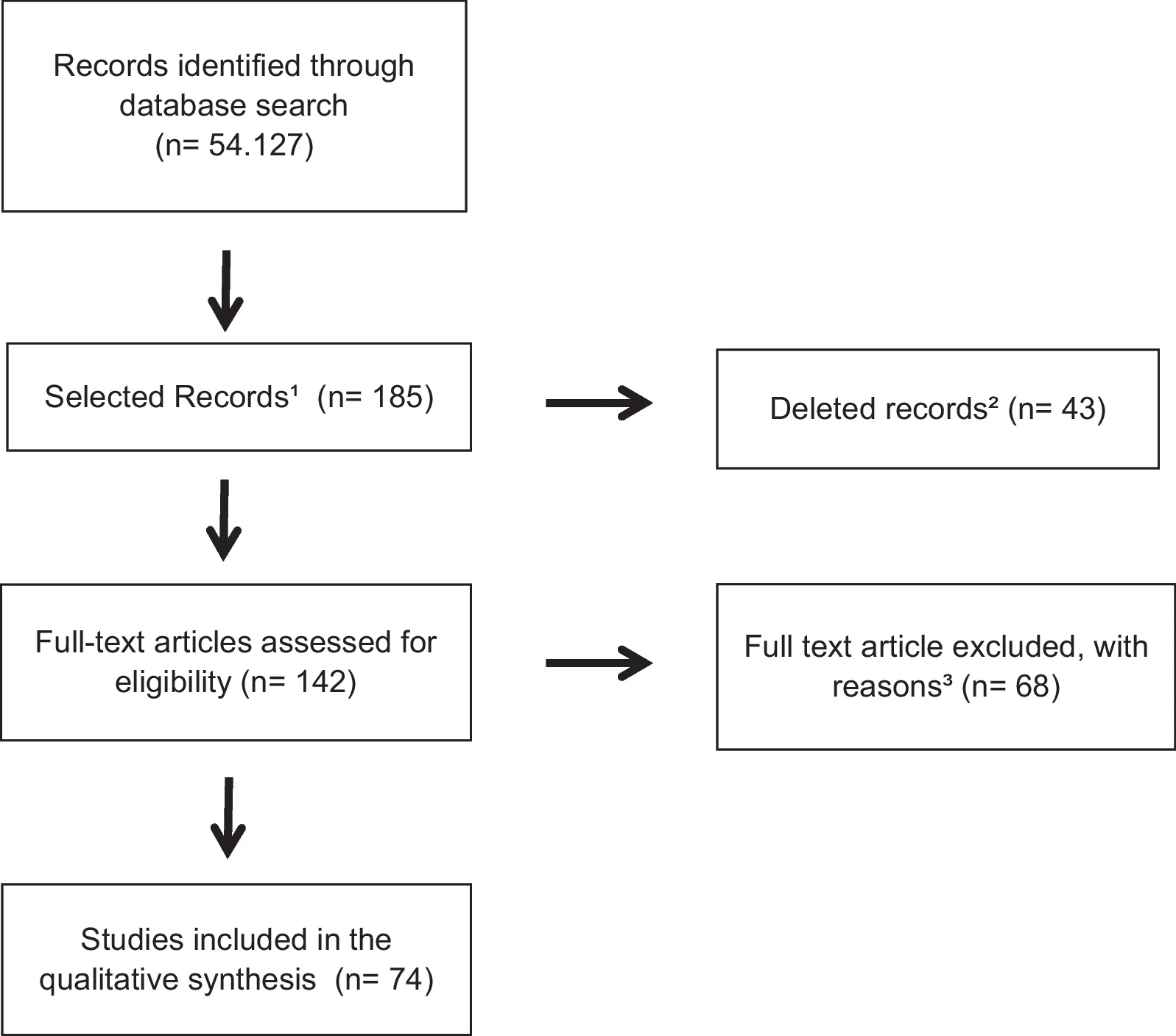
Schematic model of the information search strategy. ^1,2,3^ Full articles excluded for not being related to the topic of this article, articles with repeated information or articles not published in English and/or Portuguese

PubMed, SciELO, and Embase databases were searched for articles published from 1984 to 2022, using the following terms and combinations: “systemic lupus erythematosus”, “neuropsychiatric systemic lupus erythematosus”, “vitamin D and systemic lupus erythematosus”, “vitamin D and neuropsychiatric systemic lupus erythematosus”. The article inclusion criteria were: complete articles on the pathophysiology of SLE and NPSLE, as well as original articles that evaluated vitamin D supplementation or vitamin D levels in animal models or patients with NPSLE. The exclusion criteria for articles were: themes unrelated to the research objectives, articles whose full versions were unavailable, articles with repeat information and articles not published in English and/or Portuguese (Fig. 1).

## Neuropsychiatric lupus

Neuropsychiatric symptoms can range from relatively mild or non-specific manifestations to more severe complications. The heterogeneity and non-specificity of clinical symptoms tend to make the diagnosis difficult [3, 12, 13]. Different mechanisms have been associated with NPSLE, like blood–brain barrier (BBB) disruption, autoantibody production, proinflammatory cytokines and premature atherosclerosis. Throughout the disease course, both CNS and PNS can be affected; symptomatology in the CNS generally is divided into focal and diffuse manifestations. Focal manifestations can be partially characterized by the presence of autoantibodies targeting membrane phospholipids on the blood vessels endothelial cells in the CNS. While diffuse symptoms appear to be related to the inflammation triggered by different mediators, some of them are associated with the leakage of the BBB [3, 12].

Cohen and colleagues [14] performed a brain histopathology post-mortem study in NPSLE patients, with the majority of the lesions found in the cerebral cortex. In their study, micro and macroinfarction, microthrombi and vasculitis were more frequently found in NPSLE individuals; with microthrombi being exclusively found in NPSLE. It was noticed that in NPSLE and individuals with SLE vasculitis was found alongside diffuse vasculopathy, but focal vasculopathy was present in SLE and also in control patients. Deposits of complement components C1q and C4d, and the terminal complement complex C5B-9, were present in the cerebral blood vessels of both NPSLE and SLE patients. The accumulation of antibodies in small vessels could be what leads to the activation of the complement pathway, followed by endothelial injury and formation of microthrombi [14]. Leukocyte agglutination and accelerated atherosclerosis contribute to the thrombosis process in patients with NPSLE, and this process is involved in the pathophysiology of the disease [15]. Thrombosis can cause several complications to the patient, including focal cerebral ischemia and intracranial vascular embolism [16]. Animal models of NPSLE also demonstrate signs of depression, anxiety, cognitive deficits and brain IgG deposits [3, 17–20].

The American College of Rheumatology (ACR) classified 19 neuropsychiatric manifestations (Table 1) and recommended diagnostic methods, such as laboratory and image tests, aiming to simplify clinical cases identification and scientific research [21, 22]. NPSLE shows a higher prevalence of 91% when non-specific symptoms are included in the diagnosis, such as anxiety and headache, compared to the 46% prevalence described without them [23]. This wide variation would be associated with several factors such as population type and characteristics, the patient symptoms severity, study model, etc. [2]. Cognitive impairment, acute confused state and peripheral neuropathy would be symptoms manifested in a few SLE patients, 1 to 5%. Myelitis, aseptic meningitis and face or members involuntary movements are extremely rare to occur [1]. The difficulty presented in the identification and diagnosis remains an obstacle to establishing a clinical pattern, despite the NPSLE guidelines. It is still a major challenge without robust diagnostic tests.

**Table 1:** Neuropsychiatric syndromes observed in patients with Systemic Lupus Erythematosus

Central nervous system	Peripheral nervous system
Aseptic meningitis	Acute inflammatory demyelinating polyradiculoneuropathy
Cerebrovascular disease	(Guillain–Barre syndrome)
Demyelinating syndrome	Autonomic disorder
Headache (including migraine and benign intracranial hypertension)	Myasthenia gravis
Movement disorder (chorea)	Cranial neuropathy
Myelopathy	Plexopathy
Seizure disorders	Polyneuropathy
Acute confusional state	
Anxiety disorder	
Cognitive dysfunction	
Mood disorder	
Psychosis	

Adapted from LIANG et al. [21]

Cognitive disorder (CD) is defined as a significant deficit in any of the cognitive domains of simple and complex attention, reasoning, executive function, memory, visual-spatial processing, language, and psychomotor speed. Other factors which have been associated with CD in SLE include depression status, longer disease duration, regular glucocorticoid use and the presence of anti-neuronal and anti-phospholipid antibodies (aPLs), neuronal loss or dysfunction, and vit-D deficiency [24].

Depression and cognitive dysfunction are the most common psychiatric manifestations in SLE and were found to be present and associated in newly diagnosed SLE patients. Whether they are associated with lupus activity or medications, or are a consequence of the stress of living with a chronic disease remains debatable [25]. The signs of CD are often subtle and screening tools have been validated for the diagnosis. To date, there are no consensus guidelines for the treatment of CD in SLE [24].

PNS involvement in patients with SLE is the biggest cause of morbidity. Despite its substantial potential to impact a patient's condition, PNS involvement in SLE has not been comprehensively characterized in terms of severity, clinical associations, and electrophysiological findings. Prevalence of PNS in SLE occurs more frequently in SLE patients with high disease activity and CNS involvement, reaching approximately 8% of SLE patients [26–28].

Common PNS manifestations are chronic inflammatory demyelinating polyneuropathy, Guillain–Barré, peripheral neuropathy associated with vasculitis. There is a predilection for asymmetric and lower extremities involvement, especially peroneal, medium and sural nerves. The pathogenesis is still poorly understood. However, several factors have been identified, including vasculitis affecting small blood vessels, the presence of immune complex deposits, and lesions caused by the overproduction of antibodies or hyperactivity of B-lymphocytes [26, 29, 30].

Despite this standardized nomenclature by the ACR, the attribution of neuropsychiatric events to SLE is challenging. For correct diagnostic characterization, attribution algorithms were created, which contribute to a better characterization of the disease. Such algorithms can be translated into a probability score to determine the strength of the relationship (i.e., attribution) between a given neuropsychiatric event occurring and the underlying SLE [31–33].

The algorithm proposed by Hanly is based on three simple rules, which take into account the temporal relationship between the neuropsychiatric event and the SLE diagnosis, the type of neuropsychiatric event and a comprehensive list of exclusions/associations according to ACR nomenclature. Bortoluzzi et al. [34] added a new item to the algorithm and proposed assigning a numerical score to each selected item and its corresponding subtitle, generating a global score that varies from 0 to 10, where the higher the global score, the greater the probability whether the neuropsychiatric event can be attributed to SLE [34].

## Vitamin D

Vitamin D is a steroid hormone that can be found in several forms, being vitamin D3 (cholecalciferol) and vitamin D2 (ergocalciferol) the main types found. Cholecalciferol is formed in the skin through exposure to sunlight or ultraviolet light (Fig. 3), as well as nutrient sources such as fatty fish. Ergocalciferol is obtained from the ingestion of plant-based food. There are some known limitations for the synthesis of this vitamin, such as age, skin pigmentation, use of sunscreen and clothing [35, 36].

This steroid is widely known for its participation in the calcium metabolism [10]. In recent years, some non-classical functions of vit-D have been reported, such as effects on cell proliferation and differentiation, as well as immunoregulatory actions, resulting in the ability to maintain tolerance and promote protective immunity. Both antigen-presenting cells (macrophages and dendritic cells) and T and B cells have the necessary machinery to synthesize and respond to 1,25(OH)2D [37].

**Fig. 2:**
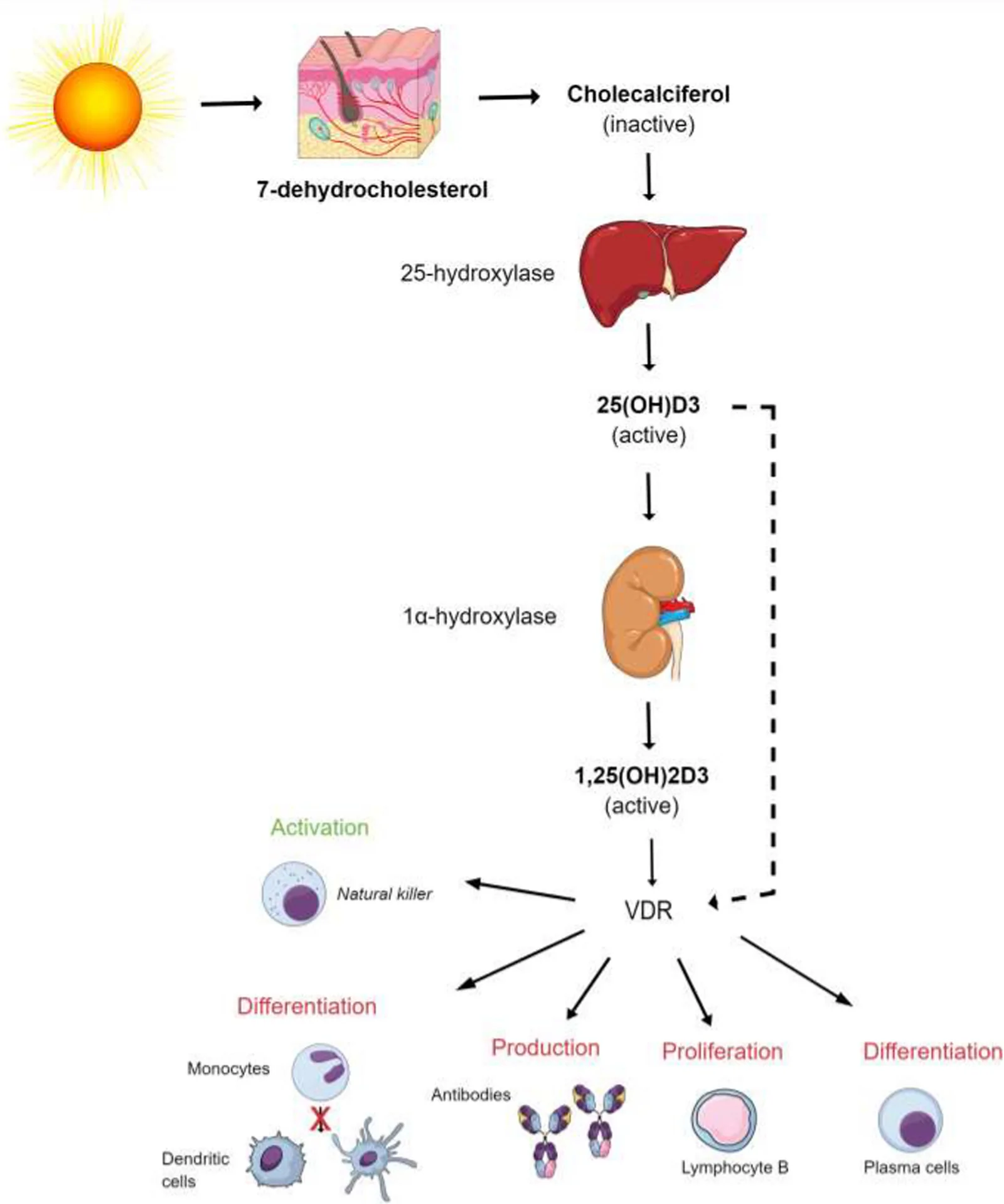
Vitamin D metabolism, activation and immunomodulatory effects. Vitamin D3 (cholecalciferol) can be obtained from food intake or by synthesis in the skin of 7-dehydroxycholesterol in response to ultraviolet (UV) light also emitted by sunlight. In the liver, hydroxylation occurs by 25-hydroxylase, forming 25-hydroxyvitamin D3 (25(OH)D3). In the kidneys, 1α-hydroxylase acts in the hydroxylation of 25(OH)D3 to the most biologically active form of vitamin D, 1,25-dihydroxyvitamin D3 (1,25(OH)2D3). Both 1,25(OH)2D3 and 25(OH)D3 are immunomodulators by binding to a vitamin D receptor (VDR) present in the nucleus of almost all immune cells. Its immunomodulatory effects include inhibition of monocyte differentiation into dendritic cells, B cell proliferation, plasma cell differentiation and antibody production. The vitamin also induces the activation of natural killer (NK) cells

Evidence suggests that vit-D has a regulatory role in the innate and adaptive immune systems. This molecule can contribute to hematopoiesis, assist in the antimicrobial response or even decrease the antigen presentation by monocytes, the latter probably having a role in maintaining immune tolerance [38, 39]. It also aids in inhibiting the differentiation and maturation of dendritic cells (DC). When mature, DCs present the antigen to T cells, facilitating an immune response against that antigen. When immature, antigen presentation facilitates self-tolerance [10]. In addition to inducing the activation of Treg cells and natural killer cells (NK), it increases apoptosis induced by dendritic cells and T lymphocytes (Fig. 2) [40]. In addition, vit-D inhibits proliferation and blocks B cell differentiation and immunoglobulin secretion [41, 42]. It also suppresses T cell proliferation and assists in inhibiting the production of inflammatory cytokines such as IL-1, IL-6, IL-17 and IL-21 and TNFα, increasing the production of anti-inflammatory cytokines such as IL-10 [10, 43, 44].

The main source of vit-D biosynthesis is exposure to sunlight. Vit-D deficiency is a consequence of low exposure to sunlight and has been associated with several physiological complications [4]. Due to the photosensitivity caused by SLE, sunlight becomes a trigger for patients with this disease [45]. Vit-D deficiency is common in SLE patients [4]. In addition, researchers have found an association between low levels of vitamin D and cognitive dysfunction in diseases such as multiple sclerosis, Alzheimer's and SLE [9, 46–48].

## Vitamin D and central or peripheral nervous system

Vitamin D expresses metabolites that are able to cross the BBB. In the human brain and in murines, vitamin D receptor (VDR) proteins are also expressed in different regions such as the cerebellum, thalamus, hypothalamus, basal ganglia, hippocampus, olfactory system and the temporal, orbital and cingulate cortices [49–51]. In some brain cells, VDR is co-localized with 1ɑ-hydroxylase enzymes. With this, there is a local conversion of vit-D to its active form (1,25 (OH)2D3 [50, 52]. Also, VDR expression in the cortex and hippocampus suggests a potential involvement of vit-D in cognition [53].

Vit-D supports brain function by contributing to the physiology of transmission and connectivity of neural circuits that are involved in reward-dependent locomotor and emotional behavior and cognition [54]. Evidence indicates an association between low serum levels of vit-D and different autoimmune and neurological diseases, as well as neuromuscular disorders and increased sensitivity to pain [55–58]. Researchers suggest the participation of vit-D in the regulatory mechanisms of the sleep–wake cycle, demonstrating that the reduction of this vitamin may be associated with sleep disorders [59, 60], as well as the perception of painful stimuli [61, 62].

**Fig. 3:**
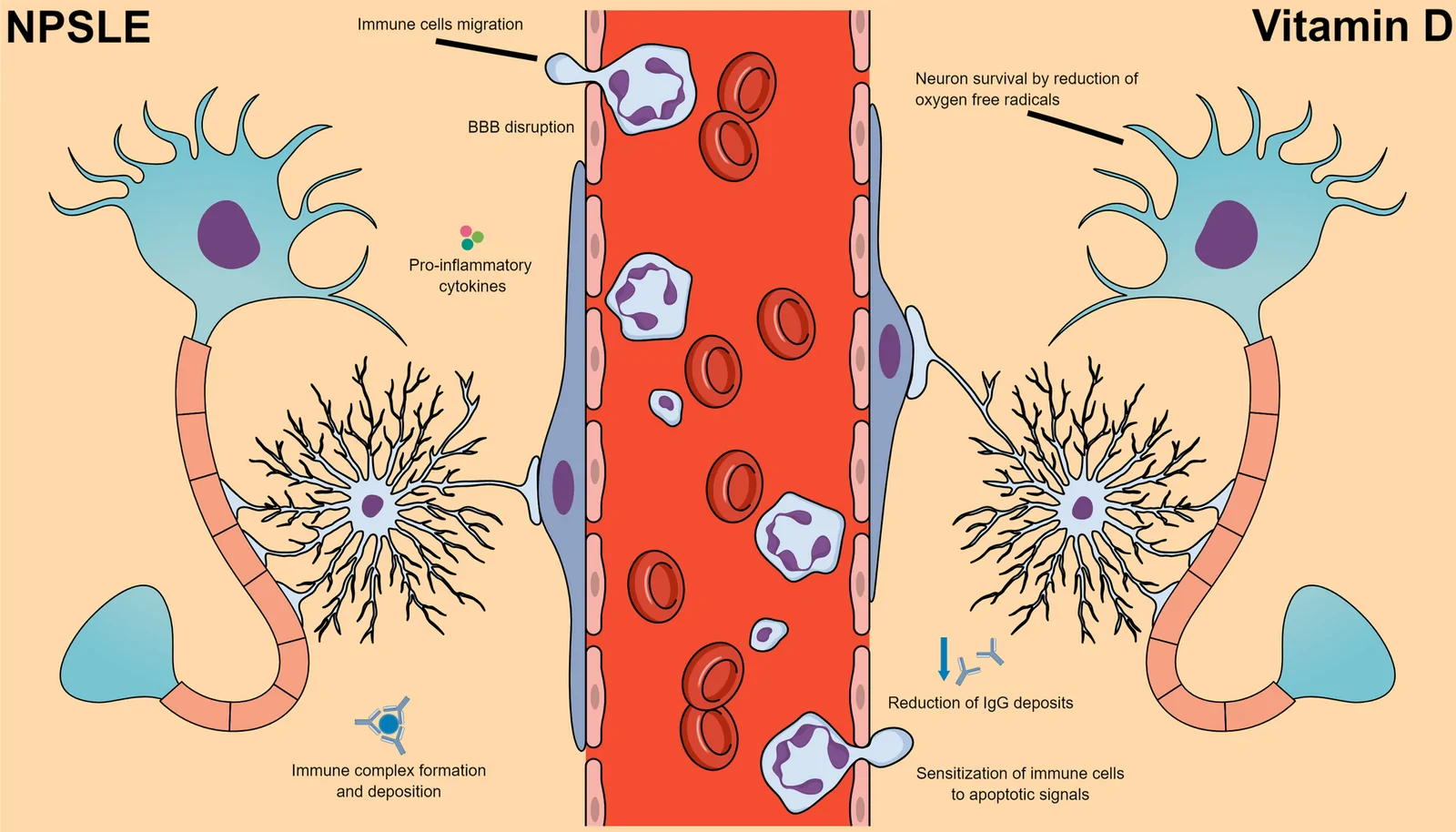
In NPSLE, the disruption of the blood–brain barrier allows the passage of cells from the immune system and molecules to the brain. Vitamin D is able to reduce free radicals in the brain environment providing neuronal survival. It is also able to reduce IgG deposits and ensure apoptotic signaling by immune system cells

This vitamin plays an important role in promoting neuron survival. It may suppress oxidative pathways in the brain by decreasing the formation of oxygen free radicals [63]. Studies show the immunomodulatory effects of vit-D in the brain (Fig. 3). Such effects can be observed on neurotrophic function, neuroprotection and neuroimmunomodulation in vitro, positive regulation of the synthesis of nerve growth factor and neurotropin 3. Furthermore, it inhibits the expression of class II proteins of the major histocompatibility complex (MHC II) and sensitizes inflammatory cells to apoptotic signals [64, 65]. In addition to these properties, 1,25(OH)2D3 is capable of regulating intraneuronal calcium homeostasis through voltage-gated calcium channels, in addition to obtaining antioxidant effects with neuroprotective properties against glutamate toxicity [64, 66].

Vit-D has also been shown to be involved in remyelination in immune-mediated diseases of the central nervous system (CNS), such as multiple sclerosis (MS). The metabolism of vit-D present in the CNS participates in myelination and can be influenced by external factors such as diet, sun exposure or vit-D supplementation [67].

## Vitamin D and NPSLE

Vit-D deficiency has been linked to cognitive dysfunction in lupus patients [68], as well as being associated with the neuropsychiatric manifestations of lupus [69] (Table 2). Studies on the effects of vit-D on NPSLE are scarce. A study carried out with MRL/lpr mice supplemented with 2 μg/kg/day once a day for four weeks demonstrated beneficial effects of supplementation. The findings revealed an improvement in the animals' cognition, as well as an increase in VDR expression in the hippocampus [70]. Our group also carried out a study to verify the effects of vitamin D supplementation (2 μg/kg once a day on alternate days for 180 days) in the hippocampus of BALB/c mice with pristane-induced lupus. Our findings revealed a positive correlation between levels of IgG deposits and VDR expression in the hippocampus [18]. Also, there is evidence in the literature that vit-D can delay cell infiltration in the choroid plexus and decrease markers suggestive of cognitive decline in MRL/lpr mice [71].

**Table 2:** Published articles relating vitamin D and neuropsychiatric manifestations in SLE

Author	Animal models			Humans		
	Yan et al. (70)	Karnopp et al. (18)	Li et al. [71]	Tay et al. [68]	Hussein et al. [48]	Sultana et al. (69)
Origin	China	Brazil	China	Singapore	Egypt	Bangladesh
Mouse model	MRL/lpr	Pristane-induced lupus	MRL/lpr	N/A	N/A	N/A
Summary	Twenty male MRL/lpr mice (aged 2 months) were divided into two groups: SLE and SLE + VD; ten male C57BL/6 J mice were used as the control group	Twenty-three female BALB/c mice (aged 2 months) were divided into three groups: control (CO), pristane-induced lupus (PIL) and PIL mice supplemented with VD (VD)	Forty-eight female MRL/lpr mice (aged 2 months) were divided into two groups: VitD3-treated group and control group. Mice were euthanized at 0 weeks (T1), 2 weeks (T2), 4 weeks (T3) and 6 weeks (T4)	Cross-sectional study in which SLE patients and healthy controls were administered Automated Neuropsychological Assessment Metrics, evaluating total throughput score (TTS) and the Hospital Anxiety and Depression Scale (HADS). Levels of 25(OH)D3 and 25(OH)D were measured	Cross-sectional, case–control study for which thirty patients diagnosed with SLE were recruited along with twenty control individuals with no history of neurologic or psychiatric conditions. All subjects were submitted to a battery of neuropsychological tests. Blood samples were collected to assess serum molecules, including vitamin D	Cross-sectional study in which 19 patients undergoing brain perfusion SPECT were retrospectively studied. All individuals had vitamin D serum level determined
Treatment	SLE-VD group was treated with *Aerococcus* and one daily intraperitoneal injection of Vitamin D (2 ug/kg/day) for 4 weeks	VD mice were supplemented with subcutaneous injections of Calcijex (2 µg/kg) every two days for 6 months	Mice in the VitD3-treated group received a 4 µg/kg 1,25-dihydroxivitamin D3 intraperitoneal injection twice a week for 3 weeks	N/A	N/A	N/A
Main outcomes	SLE + VD group presented: better performance on Morris maze test; less degeneration in the hippocampus; increased expression of VDR and Bcl-2 and decreased expression of Aβ and cleaved caspase-3 in the hippocampus	Positive correlation between VDR and IgG levels in the hippocampus of PIL and VD groups	VitD3-treated group presented: inhibited expression of genes related to cognitive dysfunction; decrease of pathological findings in brain tissues; decreased anti-dsDNA and increased C3 serum levels; increased expression of occludin and claudin-2 in the brain; increased PPAR-γ, BDNF, TGF-β1, TβR-I, Smad2/3 and P-Smad2/3 expression; decreased NF-κB and TNF-α expression	SLE patients presented more 25(OH)D_3_ deficiency than healthy controls; this deficiency predicted worse TTS; age was the only predictor of TTS	SLE patients presented significantly lower levels of serum Vit-D compared with controls; there was a negative correlation between vitamin D serum levels and performance in executive function tests in the SLE group	Positive correlation between vitamin D serum level and brain perfusion

Similar to those findings in animal models, a worsening of cognitive function was identified in humans with NPSLE and 25(OH)D3 deficiency [48, 68]. Sultana and colleagues [69] suggest, from their findings in patients, that NPSLE is associated with reduced vit-D binding protein expression in patients’ serum. Furthermore, the authors suggest that hypovitaminosis D precedes the progression of cerebral hypoperfusion in NPSLE patients and that vit-D may have prophylactic implications [69].

Hypovitaminosis D is common in patients with SLE and NPSLE, dosage adjustment must be made for each patient taking into account their clinical history and metabolism capacity. Although vit-D supplementation brings numerous benefits to the body and brain, including neuroprotection and neuroimmunomodulation, as well as improving cognition, high doses can be harmful, becoming toxic. The most common symptoms of vit-D toxicity are gastrointestinal disorders like anorexia, diarrhea, constipation, nausea, and vomiting. After a few days or weeks, other symptoms may appear, such as bone pain, drowsiness, continuous headaches, irregular heartbeat, loss of appetite, muscle and joint pain, frequent urination, especially at night, excessive thirst, weakness, nervousness and itching, and kidney stones [72].

Jones, in his review of vit-D doses and toxicity, concluded that plasma vit-D concentration is a good biomarker of toxicity. The author indicates that the threshold for toxic symptoms is approximately 750 nmol/L [73]. Although there are no reports on the effects of high doses in patients with NPSLE, a study carried out with patients with multiple sclerosis reveals that high doses of vit-D can be harmful to the CNS, promoting the worsening of demyelination [74].

## Conclusion

There is a growing scientific interest about the immunomodulatory properties of vit-D in the brain, with new articles being published in recent years in the field of NPSLE. With this review, we concatenated those studies to show that vit-D seems to have a positive association with cognitive function and beneficial effects on neuropsychiatric manifestations. Nonetheless, we believe that more studies are needed to dig deep into the questions that remain unanswered, specially about the effects of vit-D on both the central and the peripheral nervous system, either in human patients or animal models.

## Data Availability

Not applicable.

## Abbreviations

1,25(OH)2D3: 1,25-Dihydroxyvitamin D3
25(OH)D3: 25-Hydroxyvitamin D3
ACR: American College of Rheumatology
CD: Cognitive disorder
CIDP: Chronic inflammatory demyelinating polyneuropathy
CNS: Central nervous system
DC: Dendritic cells
ECs: Endothelial cells
MS: Multiple sclerosis
NK: Natural killer cells
NMO: Neuromyelitis optica
NPSLE: Neuropsychiatric lupus
MHC: Major histocompatibility complex
PNS: Peripheral nervous system
PRES: Posterior reversible encephalopathy syndrome
SFN: Small fiber neuropathy
SLE: Systemic lupus erythematosus
VDR: Vitamin D receptor
Vit-D: Vitamin D

## References

[1] Fujieda Y. Diversity of neuropsychiatric manifestations in systemic lupus erythematosus. Immunol Med. 2020;43(4):135–41. 32459601 10.1080/25785826.2020.1770947

[2] Carrión-Barberà I, Salman-Monte TC, Vílchez-Oya F, Monfort J. Neuropsychiatric involvement in systemic lupus erythematosus: a review. Autoimmun Rev. 2021;20(4):102780. 33609799 10.1016/j.autrev.2021.102780

[3] Karnopp TE, Chapacais GF, Freitas EC, Monticielo OA. Lupus animal models and neuropsychiatric implications. Clin Rheumatol. 2021;40(7):2535–45. 33155159 10.1007/s10067-020-05493-7

[4] Yap KS, Morand EF. Vitamin D and systemic lupus erythematosus: Continued evolution. Int J Rheum Dis. 2015;18(2):242–9. 25522756 10.1111/1756-185X.12489

[5] Jeffery LE, Raza K, Hewison M. Vitamin D in rheumatoid arthritis—towards clinical application. Nat Rev Rheumatol. 2015;12(4):201–10. 26481434 10.1038/nrrheum.2015.140

[6] Mathieu C. Vitamin D and diabetes: Where do we stand? Diabetes Res Clin Pract. 2015;108:201–9. 25700626 10.1016/j.diabres.2015.01.036

[7] Shoenfeld N, Amital H, Shoenfeld Y. The effect of melanism and vitamin D synthesis on the incidence of autoimmune disease. Nat Clin Pract Rheumatol. 2009;5:99–105. 19182816 10.1038/ncprheum0989

[8] Adorini L. Intervention in autoimmunity: The potential of vitamin D receptor agonists. In: Cellular immunology. 2005. p. 115–24. 10.1016/j.cellimm.2005.04.013 15936743

[9] Kamen DL. Vitamin d in lupus: New kid on the block? Bull NYU Hosp Jt Dis. 2010;68(3):218–22. 20969555 PMC4185297

[10] Aranow C. Vitamin D and the immune system. J Investig Med. 2011;59(6):881–6. 21527855 10.231/JIM.0b013e31821b8755 PMC3166406

[11] Yap KS, Northcott M, Hoi ABY, Morand EF, Nikpour M. Association of low vitamin D with high disease activity in an Australian systemic lupus erythematosus cohort. Lupus Sci Med. 2015;2(1):e000064. 25893106 10.1136/lupus-2014-000064 PMC4395813

[12] Manca E. Autoantibodies in neuropsychiatric systemic lupus erythematosus (NPSLE): Can they be used as biomarkers for the differential diagnosis of this disease? Clin Rev Allergy Immunol. 2021;63(2):194–209. 34115263 10.1007/s12016-021-08865-2 PMC9464150

[13] Govoni M, Bortoluzzi A, Padovan M, Silvagni E, Borrelli M, Donelli F, et al. The diagnosis and clinical management of the neuropsychiatric manifestations of lupus. J Autoimmun. 2016;74:41–72. 27427403 10.1016/j.jaut.2016.06.013

[14] Cohen D, Rijnink EC, Nabuurs RJA, Steup-Beekman GM, Versluis MJ, Emmer BJ, et al. Brain histopathology in patients with systemic lupus erythematosus: identification of lesions associated with clinical neuropsychiatric lupus syndromes and the role of complement. Rheumatology. 2017;56(1):77–86. 28028157 10.1093/rheumatology/kew341

[15] Thirunavukkarasu B, Gupta K, Nada R, Rathi M, Dhir V, Ahuja CK, et al. Neuropathological spectrum in systemic lupus erythematosus: A single institute autopsy experience. J Neuroimmunol. 2021;353: 577518. 33601129 10.1016/j.jneuroim.2021.577518

[16] Liu Y, Tu Z, Zhang X, Du K, Xie Z, Lin Z. Pathogenesis and treatment of neuropsychiatric systemic lupus erythematosus: a review. Front Cell Dev Biol. 2022;10:998328. 36133921 10.3389/fcell.2022.998328 PMC9484581

[17] Yun Y, Wang X, Xu J, Jin C, Chen J, Wang X, et al. Pristane induced lupus mice as a model for neuropsychiatric lupus (NPSLE). Behav Brain Funct. 2023;19(1):3. 36765366 10.1186/s12993-023-00205-y PMC9921421

[18] Karnopp TE, Freitas EC, Rieger A, Chapacais GF, Monticielo OA. Higher IgG level correlated with vitamin D receptor in the hippocampus of a pristane-induced lupus model. Clin Rheumatol. 2022;14:1859. 10.1007/s10067-022-06094-2 35149930

[19] Luciano-Jaramillo J, Sandoval-García F, Vázquez-Del Mercado M, Gutiérrez-Mercado YK, Navarro-Hernández RE, Martínez-García EA, et al. Downregulation of hippocampal NR2A/2B subunits related to cognitive impairment in a pristane-induced lupus BALB/c mice. Kavushansky A, editor. PLoS ONE. 2019;14(9):e0217190. 10.1371/journal.pone.0217190 PMC6733477 31498792

[20] Jeltsch-David H, Muller S. Neuropsychiatric systemic lupus erythematosus: Pathogenesis and biomarkers. Nat Rev Neurol. 2014;10(10):579–96. 25201240 10.1038/nrneurol.2014.148

[21] Liang MH, Corzillius M, Bae SC, Lew RA, Fortin PR, Gordon C, et al. The American College of Rheumatology nomenclature and case definitions for neuropsychiatric lupus syndromes. Arthritis Rheum. 1999;42(4):599–608. 10211873 10.1002/1529-0131(199904)42:4<599::AID-ANR2>3.0.CO;2-F

[22] Aringer M, Costenbader K, Daikh D, Brinks R, Mosca M, Ramsey-Goldman R, et al. 2019 European League Against Rheumatism/American College of Rheumatology classification criteria for systemic lupus erythematosus. Ann Rheum Dis. 2019;78(9):1151–9. 31383717 10.1136/annrheumdis-2018-214819

[23] Ainiala H, Hietaharju A, Loukkola J, Peltola J, Korpela M, Metsänoja R, et al. Validity of the new American College of Rheumatology criteria for neuropsychiatric lupus syndromes: a population-based evaluation. Arthritis Rheum. 2001;45(5):419–23. 11642640 10.1002/1529-0131(200110)45:5<419::aid-art360>3.0.co;2-x

[24] Seet D, Allameen NA, Tay SH, Cho J, Mak A. Cognitive dysfunction in systemic lupus erythematosus: immunopathology, clinical manifestations. Neuroimag Manag Rheumatol Ther. 2021;8(2):651–79. 10.1007/s40744-021-00312-0 PMC8217391 33993432

[25] Meszaros ZS, Perl A, Faraone SV. Psychiatric symptoms in systemic lupus erythematosus. J Clin Psychiatry. 2012;73(07):993–1001. 22687742 10.4088/JCP.11r07425 PMC9903299

[26] Florica B, Aghdassi E, Su J, Gladman DD, Urowitz MB, Fortin PR. Peripheral neuropathy in patients with systemic lupus erythematosus. Semin Arthritis Rheum. 2011;41(2):203–11. 21641018 10.1016/j.semarthrit.2011.04.001

[27] Hanly JG, Li Q, Su L, Urowitz MB, Gordon C, Bae S, et al. Peripheral nervous system disease in systemic lupus erythematosus: results from an international inception cohort study. Arthritis & Rheumatology. 2020;72(1):67–77. 31390162 10.1002/art.41070 PMC6935421

[28] Toledano P, Orueta R, Rodríguez-Pintó I, Valls-Solé J, Cervera R, Espinosa G. Peripheral nervous system involvement in systemic lupus erythematosus: prevalence, clinical and immunological characteristics, treatment and outcome of a large cohort from a single centre. Autoimmun Rev. 2017;16(7):750–5. 28483540 10.1016/j.autrev.2017.05.011

[29] Kieseier BC, Lehmann HC, Hörste GM. Autoimmune diseases of the peripheral nervous system. Autoimmun Rev. 2012;11(3):191–5. 21621007 10.1016/j.autrev.2011.05.011

[30] Martinez ARM, Faber I, Nucci A, Appenzeller S, França MC. Autoimmune neuropathies associated to rheumatic diseases. Autoimmun Rev. 2017;16(4):335–42. 28216073 10.1016/j.autrev.2017.02.003

[31] Hanly JG, McCurdy G, Fougere L, Douglas JA, Thompson K. Neuropsychiatric events in systemic lupus erythematosus: attribution and clinical significance. J Rheumatol. 2004;31(11):2156–62. 15517627

[32] Brey RL, Holliday SL, Saklad AR, Navarrete MG, Hermosillo-Romo D, Stallworth CL, et al. Neuropsychiatric syndromes in lupus. Neurology. 2002;58(8):1214–20. 11971089 10.1212/wnl.58.8.1214

[33] Hanly JG. ACR classification criteria for systemic lupus erythematosus: limitations and revisions to neuropsychiatric variables. Lupus. 2004;13(11):861–4. 15580983 10.1191/0961203304lu2024oa

[34] Bortoluzzi A, Scirè CA, Bombardieri S, Caniatti L, Conti F, De Vita S, et al. Development and validation of a new algorithm for attribution of neuropsychiatric events in systemic lupus erythematosus. Rheumatology. 2015;54(5):891–8. 25339643 10.1093/rheumatology/keu384

[35] Lips P. Vitamin D physiology. Prog Biophys Mol Biol. 2006;92:4–8. 16563471 10.1016/j.pbiomolbio.2006.02.016

[36] Chang SW, Lee HC. Vitamin D and health - The missing vitamin in humans. Pediatr Neonatol. 2019;60(3):237–44. 31101452 10.1016/j.pedneo.2019.04.007

[37] Wu S, Ren S, Nguyen L, Adams JS, Hewison M. Splice variants of the CYP27b1 gene and the regulation of 1,25-dihydroxyvitamin D3 production. Endocrinology. 2007;148(7):3410–8. 17395703 10.1210/en.2006-1388

[38] Bellan M, Andreoli L, Mele C, Sainaghi PP, Rigamonti C, Piantoni S, et al. Pathophysiological role and therapeutic implications of vitamin D in autoimmunity: focus on chronic autoimmune diseases. Nutrients. 2020;12(3):789. 32192175 10.3390/nu12030789 PMC7146294

[39] Medrano M, Carrillo-Cruz E, Montero I, Perez-Simon J. Vitamin D: effect on haematopoiesis and immune system and clinical applications. Int J Mol Sci. 2018;19(9):2663. 30205552 10.3390/ijms19092663 PMC6164750

[40] Pelajo CF, Lopez-Benitez JM, Miller LC. Vitamin D and autoimmune rheumatologic disorders. Autoimm Rev. 2010;9:507–10. 10.1016/j.autrev.2010.02.011 20146942

[41] Lemire JM, Adams JS, Sakai R, Jordan SC. 1 alpha,25-dihydroxyvitamin D3 suppresses proliferation and immunoglobulin production by normal human peripheral blood mononuclear cells. J Clin Investig. 1984;74(2):657–61. 6611355 10.1172/JCI111465 PMC370520

[42] Chen S, Sims GP, Chen XX, Gu YY, Chen S, Lipsky PE. Modulatory effects of 1,25-dihydroxyvitamin D 3 on human B cell differentiation. J Immunol. 2007;179(3):1634–47. 17641030 10.4049/jimmunol.179.3.1634

[43] Bhalla AK, Amento EP, Serog B, Glimcher LH. 1,25-Dihydroxyvitamin D3 inhibits antigen-induced T cell activation. J Immunol (Baltimore, Md: 1950). 1984;133:1748–54. 6206136

[44] Almerighi C, Sinistro A, Cavazza A, Ciaprini C, Rocchi G, Bergamini A. 1α,25-Dihydroxyvitamin D3 inhibits CD40L-induced pro-inflammatory and immunomodulatory activity in Human Monocytes. Cytokine. 2009;45(3):190–7. 19186073 10.1016/j.cyto.2008.12.009

[45] Hassanalilou T, Khalili L, Ghavamzadeh S, Shokri A, Payahoo L, Bishak YK. Role of vitamin D deficiency in systemic lupus erythematosus incidence and aggravation. Auto-Immunity Highlights. 2018;9(1):1. 10.1007/s13317-017-0101-x PMC5743852 29280010

[46] Ginzler E, Tayar J. Systemic lupus erythematosus. American College of Rheumatology. 2013;1–6.

[47] Schwartz N, Stock AD, Putterman C. Neuropsychiatric lupus: new mechanistic insights and future treatment directions. Nat Rev Rheumatol. 2019;15(3):137–52. 30659245 10.1038/s41584-018-0156-8 PMC8023338

[48] Hussein HA, Daker LI, Fouad NA, Elamir A, Mohamed SR. Does Vitamin D deficiency contribute to cognitive dysfunction in patients with systemic lupus erythematosus? Innov Clin Neurosci. 2018;15(9–10):25–9. 30588363 PMC6292718

[49] Prüfer K, Veenstra TD, Jirikowski GF, Kumar R. Distribution of 1,25-dihydroxyvitamin D3 receptor immunoreactivity in the rat brain and spinal cord. J Chem Neuroanat. 1999;16(2):135–45. 10223312 10.1016/s0891-0618(99)00002-2

[50] Eyles DW, Smith S, Kinobe R, Hewison M, McGrath JJ. Distribution of the vitamin D receptor and 1 alpha-hydroxylase in human brain. J Chem Neuroanat. 2005;29(1):21–30. 15589699 10.1016/j.jchemneu.2004.08.006

[51] Pardridge WM, Sakiyama R, Coty WA. Restricted transport of Vitamin D and A derivatives through the rat blood–brain barrier. j Neurochem. 1985;44(4):1138–41. 3838342 10.1111/j.1471-4159.1985.tb08735.x

[52] McGrath J, Feron F, Eyles D, Mackay-Sim A. Vitamin D: the neglected neurosteroid? Trends Neurosci. 2001;24(10):570–1. 11576669 10.1016/s0166-2236(00)01949-4

[53] Stewart A, Wong K, Cachat J, Elegante M, Gilder T, Mohnot S, et al. Neurosteroid vitamin D system as a nontraditional drug target in neuropsychopharmacology. Behav Pharmacol. 2010;21(5–6):420–6. 20571365 10.1097/FBP.0b013e32833c850f

[54] Bivona G, Gambino CM, Iacolino G, Ciaccio M. Vitamin D and the nervous system. Neurol Res. 2019;41:827–35. 10.1080/01616412.2019.1622872. 31142227 10.1080/01616412.2019.1622872

[55] Orme RP, Bhangal MS, Fricker RA. Calcitriol imparts neuroprotection in vitro to midbrain dopaminergic neurons by upregulating GDNF expression. PLoS ONE. 2013;8(4):e62040. 23626767 10.1371/journal.pone.0062040 PMC3633905

[56] Osunkwo I, Hodgman EI, Cherry K, Dampier C, Eckman J, Ziegler TR, et al. Vitamin D deficiency and chronic pain in sickle cell disease. Br J Haematol. 2011;153(4):538–40. 21275953 10.1111/j.1365-2141.2010.08458.x

[57] Durmer JS, Dinges DF. Neurocognitive consequences of sleep deprivation. Semin Neurol. 2005;25(01):117–29. 15798944 10.1055/s-2005-867080

[58] Adorini L, Penna G. Control of autoimmune diseases by the vitamin D endocrine system. Nat Clin Pract Rheumatol. 2008;4(8):404–12. 18594491 10.1038/ncprheum0855

[59] McCarty DE, Reddy A, Keigley Q, Kim PY, Marino AA. Vitamin D, race, and excessive daytime sleepiness. J Clin Sleep Med. 2012;08(06):693–7. 10.5664/jcsm.2266 PMC3501666 23243403

[60] Mete T, Yalcin Y, Berker D, Ciftci B, Guven SF, Topaloglu O, et al. Obstructive sleep apnea syndrome and its association with vitamin D deficiency. J Endocrinol Invest. 2013;36(9):681–5. 23558409 10.3275/8923

[61] Le Goaziou MF, Kellou N, Flori M, Perdrix C, Dupraz C, Bodier E, et al. Vitamin D supplementation for diffuse musculoskeletal pain: Results of a before-and-after study. Eur J General Pract. 2014;20(1):3–9. 10.3109/13814788.2013.825769 24576123

[62] Heidari B, Shirvani JS, Firouzjahi A, Heidari P, Hajian-Tilaki KO. Association between nonspecific skeletal pain and vitamin D deficiency. Int J Rheum Dis. 2010;13(4):340–6. 21199469 10.1111/j.1756-185X.2010.01561.x

[63] Grimm MOW, Thiel A, Lauer AA, Winkler J, Lehmann J, Regner L, et al. Vitamin D and its analogues decrease amyloid-β (Aβ) formation and increase Aβ-degradation. Int J Mol Sci. 2017;18(12):2764. 29257109 10.3390/ijms18122764 PMC5751363

[64] Annweiler C, Schott AM, Berrut G, Chauviré V, le Gall D, Inzitari M, et al. Vitamin D and ageing: neurological issues. Neuropsychobiology. 2010;62(3):139–50. 20628264 10.1159/000318570

[65] Etgen T, Sander D, Bickel H, Sander K, Förstl H. Vitamin D deficiency, cognitive impairment and dementia: a systematic review and meta-analysis. Dement Geriatr Cogn Disord. 2012;33(5):297–305. 22759681 10.1159/000339702

[66] Annweiler C, Fantino B, Parot-Schinkel E, Thiery S, Gautier J, Beauchet O. Alzheimer’s disease–input of vitamin D with mEmantine assay (AD-IDEA trial): study protocol for a randomized controlled trial. Trials. 2011;20:12. 10.1186/1745-6215-12-230 PMC3212921 22014101

[67] Matías-Guíu J, Oreja-Guevara C, Matias-Guiu JA, Gomez-Pinedo U. Vitamin D and remyelination in multiple sclerosis. Neurología (English Edition). 2018;33(3):177–86. 10.1016/j.nrl.2016.05.001 27321170

[68] Tay SH, Ho CS, Ho RCM, Mak A. 25-Hydroxyvitamin D3 deficiency independently predicts cognitive impairment in patients with systemic lupus erythematosus. Crispin J, editor. PLoS ONE. 2015;10(12):e0144149. 10.1371/journal.pone.0144149 PMC4670220 26636681

[69] Sultana N, Sarkar AK, Matsuda H, Ihsan MA, Haq SA, Arefin MS, et al. Relationship between Vitamin D status and brain perfusion in neuropsychiatric lupus. Nucl Med Mol Imaging. 2022;56(3):158–68. 35607635 10.1007/s13139-022-00741-x PMC9123148

[70] Yan L, Wu P, Gao DM, Hu J, Wang Q, Chen NF, et al. The impact of Vitamin D on cognitive dysfunction in mice with systemic lupus erythematosus. Med Sci Monit. 2019;25(25):4716–22. 31281179 10.12659/MSM.915355 PMC6607939

[71] Li X, Xu S, Liu J, Zhao Y, Han H, Li X, et al. Treatment with 1,25-Dihydroxyvitamin D3 Delays Choroid Plexus Infiltration and BCSFB Injury in MRL/lpr Mice Coinciding with Activation of the PPARγ/NF-κB/TNF-α Pathway and Suppression of TGF-β/Smad Signaling. Inflammation. 2022. 10.1007/s10753-022-01755-5 36269513

[72] Schwalfenberg G. Not enough vitamin D: health consequences for Canadians. Can Fam Physician. 2007;53(5):841–54. 17872747 PMC1949171

[73] Jones G. Pharmacokinetics of vitamin D toxicity. Am J Clin Nutr. 2008;88(2):582S-586S. 18689406 10.1093/ajcn/88.2.582S

[74] Häusler D, Torke S, Peelen E, Bertsch T, Djukic M, Nau R, et al. High dose vitamin D exacerbates central nervous system autoimmunity by raising T-cell excitatory calcium. Brain. 2019;142(9):2737–55. 31302671 10.1093/brain/awz190 PMC6736324

